# Collectanea Medica

**Published:** 1813-08

**Authors:** 


### COLLECTANEA MEDICA,

CONSISTING OF

### ANECDOTES, FACTS, EXTRACTS, ILLUSTRATIONS, QUERIES, SUGGESTIONS, &c.

RELATING TO THE

### *History or the Art of Medicine, and the Auxiliary Sciences.*

### *On the medical Effects of Climates.*

A COMPLETE system of meteorology, even so far as the properties of climates, with regard to temperature only, are concerned, presents almost as great difficulties as a complete theory of the nature and cure of diseases. In this, as in many other departments of medical knowledge, we perpetually find a multiplicity of accounts, apparently well attested, but totally at variance with each other, which render it desirable to appeal to some more satisfactory testimonials than the results of common and superficial observation; while the evidence which would be required for forming useful conclusions, upon safe and scientific grounds, although in this case completely within the scope of the human faculties, is still such as to require, for its production, a combination of perseverance and accuracy which has certainly never yet existed, and which, probably, can scarcely ever be expected to be found in a sufficient number of collateral observers. Any voluminous work on the subject, whether systematic or empirical, must unavoidably contain much useless and some erroneous matter; and a short statement of a few facts, which appear to be tolerably well ascertained, first, respecting the physical characters, and secondly, respecting the medical effects of the principal climates which deserve our notice, is all that it will be possible to attempt in the present essay.

The simple indications of a thermometer, however accurately they may be observed, in the most unexceptionable exposure, by no means afford a correct test of the temperature, as it affects the human system; nor is it possible to express the modifications produced by wind and moisture, even supposing them to be easily known, by any numerical measure which shall be applicable to every relative situation of the individual. I have known an atmosphere at 65°, with a thick fog, and a very little wind from the N. E., appear, to a person taking moderate exercise, most oppressively sultry; although a person, sitting long still, might have felt the the same air uncomfortably cold. Moisture must make both heat and cold more sensible; the one, by diminishing perspiration, the other, by increasing the conducting power of air. Wind is doubly concerned in affecting the properties of a climate; first, as the great cause of preventing a general accumulation of heat over considerable tracts of country; and secondly, as having a similar effect with respect to the immediate neighbourhood of the person; and its operation is as generally perceptible in the latter way, where we have no precise mode of estimating its magnitude, as in the former, where it is correctly indicated by a thermometer sufficiently exposed: although, in fact, the most shaded fixed thermometer may often be observed to indicate a temperature many degrees higher than that of the breeze which is circulating in the neighbouring country. Still more commonly by the sea side, the wind exhibits the temperature of the water over which it has blown. At Worthing it is seldom above 64° in the hottest weather, although the sea, when the tide flows in at noon, over the heated expanse of sand, is sometimes raised to 78°, where it is several feet deep.

To the inhabitants of these islands, the most important properties of the climates of other countries are those which render them more or less fit for the residence of persons liable to catarrhal or consumptive affections. Hence, warmth and equability of temperature, especially in the winter months, are the first objects of our inquiry in the theoretical comparison of climates. Moisture is supposed, by some, to be favorable, by others, to be unfavorable, to such persons: it may, therefore, be safely neglected, except as tending to increase the evils depending on a want of equability of temperature. The effluvia of moist ground are sufficiently well known as the causes of paludal fevers; further than this they require no particular investigation. Nor can we attempt to assign any reason for peculiarities, which render some situations preferable to others, for some individuals only, laboring under a given disease, as asthma; which is sometimes induced by the atmosphere of cities, and sometimes of the country; and which is occasionally mitigated by a residence in places having no marked distinctions from such as are less favorable to it, as Kensington, and perhaps some others.

In the hotter seasons, there are few diseases, and few constitutions, which would require a climate milder than our own: in the colder, an increase of the facility of circulation, which heat appears to afford, may often be beneficial, ficial, partly perhaps as exciting perspiration, and partly as preventing too great a congestion of blood in the internal parts of the body. The mean temperature of the six winter months is, therefore, the first point of comparison that requires our attention, and such a comparison may easily be derived from the registers, which are usually kept in circumstances nearly similar.

*From October to March.*

| | |
|---|---|
| London, R. S. 1790-4 | 43·5° |
| Edinburgh | 40·4 |
| Dawlish, Sir W. W. M. S. 1794 (Lond. 44·1°) | 45·3 |
| Ilfracombe, without doubt incorrect | (55) |
| Paris | 41·2 |
| Lisbon | 55·5 |
| Malta, Domeier | 63 |
| Madeira, Gourlay. (S.W. aspect, M.) | 63 |
| Bermudas, M.S.R.S. 1790 | 68 |
| Jamaica, Botanic garden at Kingston, Clarke, Dunc. med. comm. vii. 369 | 74·5 |

*From November to March.*

| | |
|---|---|
| London, 1808-9 | 42·6° |
| Penzance, 1808-9, Stirling, at 10, or about 1° above the mean | 48·1 |

*From January to March.*

| | | | |
|---|---|---|---|
| London, 1809 | 43·1° | (Jan. 37·9° | |
| Glasgow, 1809, Stirling, at 10 | 40·3 | 33·1 | |
| Penzance, 1809, Stirling, at 10 | 48·5 | 46·7 | (Dec. 43·7°) |
| London, 1790-4, 8 or 7 and 2 | 41·6 | 39·1 | |
| Sidmouth, 1800, M. S. R. S. 8 and 2 | 41·7 | 42·3) | |

*February and March.*

| | |
|---|---|
| London, 1803, 7 and 2 | 41·5° |
| Clifton, 1803, 8 and 2. Carrick | 42·5 |

*From October to December.*

| | |
|---|---|
| London, 1811, mean of extremes in each month | 47·0° |
| Sidmouth, 1811, Clarke | 45·7 |

*From December to February.*

| | |
|---|---|
| London | 39·7° |
| Edinburgh | 36·7 |
| Paris | 36·8 |

It appears from this comparison, that none of the situations here enumerated, North of Lisbon, except Penzance, has any material advantage over London in the mildness of its winter. The best parts of Devonshire seem to be about

 a degree a degree and a half warmer; Torquay, however, may, perhaps, be a little milder than this; the account which was kept at Ilfracombe must have been taken from a thermometer in a confined or a sunny situation. But Penzance may be fairly considered as having a temperature 4½° higher than London in the coldest months; nor is the journal here employed the only one which allots such a superiority to the climate of this extremity of our island. It is remarkable that the temperature of the three coldest months is the same at Paris as at Edinburgh, being, in both these cities, about three degrees lower than in London. There are, probably, particular spots on the coast of Hampshire or Sussex, which, from their sheltered situation, must be considerably less subject to the effect of the northern and eastern winds, than most other parts of the island; and Hastings, or its neighbourhood, may, perhaps, be reckoned among the most eligible of these; but the further we go up the channel, the more remote we become from the mild gales of the Atlantic, while the prevalent south-westerly winds, in passing over a considerable part of the continent, must have lost much of their warmth. It is scarcely necessary to observe, that both Malta and Madeira present, numerically, a mean temperature for the winter months, as favorable for an invalid as can possibly be desired.

Equability of temperature is a second quality, of no small importance, as tending to diminish the chance of incurring, or aggravating, pulmonary diseases, by repeatedly taking cold. When, indeed, the temperature is much below 60°, the most material changes are those which occur upon going from the house into the open air; so that a cold climate becomes, in some degree, of necessity a changeable one also. The regularity of this change, and the power of avoiding its effects by additional clothing, as well as of obviating them in some measure by exercise, contribute, however, to lessen its influence; and it does not, therefore, altogether supersede the effects of that changeableness, which consists in a great extent of variation of the temperature of two successive days, or of different hours in the course of the same day. The simplest, and, perhaps, the best mode of appreciating the effect of the extent of such a variation, in deteriorating a climate, is to observe, for each month, the greatest variation, at the same hour, in any two successive days within its duration. The mean variation of successive days may also be computed, in order to assist in the comparison; and the mean diurnal range, or the space through which the surface of the mercury moves, in ascending ascending and descending, throughout the day and night, will give a collateral estimate of a similar nature. The best practical mode of deducing this range from the observations is, to find separately the mean of the heights for the morning and afternoon, and to double their difference. Where none of these particulars can be obtained, the extreme variation of each month will afford a character not altogether unimportant.

*Mean of the greatest Variations of successive Days in each Month, for the Winter Months.*

| | |
|---|---|
| London, 1790–4, 6 mo. | 11·5° |
| London, 1794 (greatest of all 15°) | 10·7 |
| Knightsbridge, Read, 1790–1 (greatest 23°) | 16·3 |
| Dawlish, 1794 (greatest 13½°) | 10·7 |
| Lisbon, 1788 (greatest 11°) | 8·7 |
| Bermudas, 1790 (greatest 13°) | 9·0 |
| Montreal, 1788 | 4·0 |
| Penzance, 1808–9. Nov. to March (gr. 10°) | 9·2 |
| Sidmouth, 1800. Jan. to March (gr. 16°) | 10·9 |
| Gravesend, 1787. Jan. | 13·0 |
| Ashover, Derbyshire, 1805. Jan. | 13·5 |
| Minehead, Atkins, 1782. Jan. | 16· |
| Clifton, Feb. 1803, 9°, March, 13°, mean | 11· |

*Mean Variation of successive Days for the Winter Months.*

| | |
|---|---|
| London, 1790–4, 6 mo. | 3·62° |
| London, 1794 | 3·51 |
| Knightsbridge, 1790–1 | 5·45 |
| Dawlish, 1794 | 3·68 |
| Lisbon, 1788 | 2·70 |
| Bermudas, 1790, about | 3·00 |
| Montreal, 1778 | 13·2 |
| Penzance, 1808–9. Nov. to March | 2·80 |
| Sidmouth, 1800. Jan. to March | 3·32 |
| Clifton, 1808. Feb. and March | 3·55 |
| Gravesend, 1787. Jan. | 4·15 |
| Ashover, 1805. Jan. | 3·33 |
| Minehead, 1782. Jan. | 4·00 |

*Mean diurnal Range for the Winter Months.*

| | |
|---|---|
| London, 1790–4, 6 mo. | 13·0° |
| Sidmouth, 1800. Jan. to March | 10·0 |
| Clifton, 1808. Feb. and March (Lond. 16·2°) | 11·4 |

*Mean monthly Variation for the Winter Months.*

| | |
|---|---|
| London, 1793–6, 6 mo. | 25·9° |
| Madeira, 1793–6, 6 mo. | 12·6 |
| Sidmouth, 1811. Jan. to March | 34· |
| Clifton, 1803. Feb. and March (Lond. 36°) | 31· |

It

It does not appear that Devonshire possesses any decided advantages over London with respect to equability of climate, if we judge of the climate of London from the observations made at the apartments of the Royal Society only: but in so central a situation, the changes must be rendered much less sensible by the effect of the surrounding buildings; and they appear to be considerably greater at Gravesend, and greater still at Knightsbridge. In this respect, too, Penzance retains its superiority even over Devonshire. Lisbon seems to have a less variable temperature than any part of Great Britain; and in Madeira, to judge by the monthly variation only, the advantage in this respect appears to be still greater.

The greatest possible equability of temperature seems however to be obtained in a sea voyage to a warm climate, in which the variation seldom amounts to half as much as in the most favorable situation on shore, even on a small island; and in pulmonary cases the motion of a ship would probably, in general, be rather beneficial than otherwise, while the fatigue of travelling in bad roads, and the danger of sleeping in damp beds, present an alternative by no means favorable to a journey by land.

The direction of the wind alone can seldom have any immediate effect on the salubrity of the climate, except by variously modifying its temperature, according to the seas or countries over which it blows. There is a method of computing the mean direction of the wind, which does not appear to have been hitherto adopted, but which affords a very simple and intelligible result, although somewhat laborious if extensively applied. It consists in finding the bearing and distance of a point, to which a light body would be carried by the wind in the course of the year, supposing the velocity to be constant, when its variations have not been ascertained by observation. It is obvious that the bearing of such a point will show at once the mean direction of the prevalent winds; and its distance, compared with the effect of a constant wind for the same time, as a unit, will indicate the degree in which those winds have prevailed.

*Prevalence of Winds.*

| | | | | | | |
|---|---|---|---|---|---|---|
| London, | 1790–4 | W. | 9° | S. | ·234. |
| London, | 1794 | W. | 33° | S. | ·188. |
| Dawlish, | 1794 | W. | 6° | S. | ·466. |
| Lisbon, | 1788 | N. | 1° | W. | ·315. |

According to this comparison, it appears that the mean direction of the wind in Devonshire is somewhat more westerly than in London: and that the degree, in which such westerly westerly winds predominate, is more than twice as great as in London; or, if we convert the measure into days, that the predominance amounted, in 1794, to 68 days for London, of a wind nearly W.S.W. and to 170 days for Dawlish, of a wind a little to the south of west.

The variations of the climate of the same place, with respect to mean temperature, are easily collected from the usual meteorological computations. Dr. Heberden has very successfully combated the common opinion respecting the superior salubrity of cold winters; it appears, however, that the winter which he particularly observed was more variable, as well as colder, than usual. Mr. Kirwan has attempted to account for the greater frequency of colds, which he supposes to occur in spring and in autumn, by the greater variability of the temperature at those seasons: but both the fact and the explanation are very questionable; for in reality the variations of temperature, if estimated by the total range of the thermometer within the 24 hours, are almost uniformly greatest in the hottest weather. In London, the greatest variations of successive days at the same hours in the morning are greatest in winter; in the afternoon, in summer; and, although the latter are a little greater in April than in some of the succeeding months, the difference is by no means considerable.

Of the empirical evidence, which may be collected, respecting the medical effects of different climates, the most authentic is perhaps that which is derived from well-regulated bills of mortality; since these documents ought to afford us a tolerable criterion of the general healthiness or unhealthiness of a place, from the proportion between the annual deaths and the population, and at the same time a pretty correct determination of the degrees in which different diseases are fatal. Thus, when we find that in Stockholm the annual deaths amount to $\frac{1}{19}$ of the population, in London to $\frac{1}{21}$, in the Pays de Vaud to $\frac{1}{45}$, and in some villages in different parts of Great Britain to $\frac{1}{60}$ only, we cannot hesitate to consider a residence in the country as generally more healthy, than in a metropolis similar to either of those cities; although it cannot fairly be concluded that the healthiness is precisely in the proportion which might be inferred from this comparison, until we have considered how far the effect of emigration to a great town may influence the apparent mortality. After the age of eight or ten, the probable duration of life may be estimated with sufficient accuracy, as Demoivre has very ingeniously shown, by assuming that, of a certain number of persons born together, one will die annually until the whole number is become come extinct; and it is well known, that this number may in common cases be supposed to be 86; so that at any given age, for instance 36, we may find the probable duration of life by deducting it from 86, and halving the remainder, which will give us 25 for the estimate required; and if this law were universally true from the time of birth, it is easy to show that the mortality in a metropolis would always be increased by the accession of settlers; so that if, for example, the whole population were supplied by settlers at 20, and all children were sent to a neighbouring village to be educated, the mortality of the town, instead of $\frac{1}{43}$, would become $1 : (43 - 10) = \frac{1}{33}$, and that of the village would be $1 : (86 - 10) = \frac{1}{76}$; and that any partial changes of a similar nature would cause a smaller alteration of the apparent salubrity, in proportion to their extent. But the mortality during infancy is actually much greater than is assumed in the simple hypothesis of Demoivre; and from this circumstance, as well as from the frequent return of aged persons into the country, Dr. Price has inferred that emigration in general has no tendency to increase the mortality of cities. In reality, the question depends altogether upon the mortality which may be supposed to take place within the first year, which is often estimated at one-third of the births; but nothing like this can well be expected to occur at any tolerably healthy place in the country; and on the whole it does not appear that Dr. Price's observations can by any means be admitted as conclusive. With respect to the evidence afforded by the prevalence of diseases, it has been observed by Dr. Gregory, that removing from a colder to a warmer climate may be beneficial, even in those diseases to which the inhabitants of the warmer climate are subject; but if they appeared to be equally or more subject to any disease than the inhabitants of the colder, there would surely be little encouragement for the change: for instance, in a person supposed to be liable to diseases of the liver, it would surely be injudicious to undertake a voyage to a hot climate, with a view of avoiding the chance of taking cold, since the well-known frequency of hepatitis, in such climates, would much more than counterbalance any prospect of advantage from the change.

The frequency of consumptions is decidedly greater in cold than in hot climates, but not by any means in exact proportion to the depression of the mean temperature. The principal situations, that require to be compared with the metropolis, as a standard, are the south of England, the south of Europe, the islands of the Mediterranean, Madeira, and the West Indies.

There do not appear to be any precise accounts of the proportionate mortality from consumption at any place upon the southern coasts of this island, on a scale sufficiently extensive for the comparison; but there is abundant reason to think that such a report would be greatly in favor of the salubrity of these coasts, more so indeed than any conclusions, that we should be at all authorised to form, from such thermometrical observations as have hitherto been compared. A greater number of registers is still wanting to obtain sufficient evidence for the inquiry: and it would be desirable that some journal should be kept at one of the Scilly islands, as a situation fully exposed to the influence of the sea air; for there can be little doubt that, for equability of temperature, a very small island must have great advantages above every other situation on shore. But in the present state of our knowledge on this subject, although we are fully justified in recommending a residence in Devonshire or Cornwall as advisable in a certain stage of consumption, it does not appear that any meteorological observations will authorise us to represent the advantages to be gained by such a residence, as by any means equivalent to those which may be found in remoter situations; nor that the empirical testimony, derived from accounts of the comparative prevalence of the disease, is at all so clear, or so firmly established, as to make up for the want of evidence of a great and decided superiority of the climate.

In the south of Europe, the situations which have been most frequented are Lisbon, or some other part of the peninsula, the neighbourhood of Montpelier, and different parts of Italy. In Spain, and probably in Portugal, consumption is said to be not common, but by no means wholly unknown; and whether from accident, or from causes which are likely to have a constant operation, the climate of Portugal has certainly failed, in a number of instances, of producing any material benefit, where there has been apparently a very fair chance for the patient's recovery. With respect to the south of France, it is perhaps sufficient to remark, that the general proportion of deaths from consumption at Marseilles is fully as great, as the greatest which has been observed in London, where, according to Dr. Heberden's remark, its prevalence has of late years been so much increased. In Italy the disease appears to be decidedly less frequent; and there is no reason to doubt but that, in the southern parts of that country, there may be situations approaching in their climates to those of the neighbouring islands.

It is, however, highly probable that some of these islands possess very considerable advantages over almost every part of the continents which surround them, at least as far as we can judge by their affording a climate of that description which seems to be the most desirable; for actual experience will not allow us to be too confident of obtaining success, even from a residence in these. Dr. Domeier informs us, in his very interesting account of the island of Malta, that the thermometer seldom varies here more than 6° in the 24 hours, or stands below 51°, even in the depth of winter; while in Lisbon he has seen ice, and both ice and snow in Naples; besides that, in these two cities, the difference between day and night often amounts to 20°. If an invalid leaves England in the middle of August, the voyage lasts about a month, and is often of itself highly beneficial, so that he arrives at Malta in time to be fully prepared to be further benefited by the mild winter: it appears, however, from the more particular account which Dr. Domeier elsewhere gives of the temperature, that it continues throughout October rather higher than is altogether desirable, being seldom below 70° throughout that month; and in a country where there is scarcely any visible foliage, walls occupying universally the place of hedges, this cannot be a matter of perfect indifference.

In Madeira, though the thermometer attached to a building is seldom found below 54°, there are frequently cold winds, snow, or more commonly something intermediate between snow and hail, often falling on the mountains, at the height of 1000 feet above the sea, and at still greater elevations sometimes lying undissolved till July: and this imperfect kind of hail falls occasionally even on the low grounds. The island is probably a more agreeable residence than Malta: but it seems very doubtful whether it possesses any determinate advantage over it with respect to climate; and it is not impossible, that some other islands in its neighbourhood may afford a greater equability of temperature. We have, however, a more established experience of its beneficial effects in pulmonary diseases than of almost any other situation. Dr. Adams says, that, "in cases of tubercular or scrofulous consumption, if the patient does not saunter away his time after you have advised him to leave England, we can with certainty promise a cure." (Med. Phys. Journ. Apr. 1800.) This true English consumption he thinks is not to be found in Madeira, while the catarrhal affection, which somewhat resembles it, though without purulent expectoration, is not uncommon, and may be fatal if neglected or improperly treated. Dr. Gourlay agrees with Dr. Adams, in his report of the general benefit derived from the climate of Madeira, by consumptive persons going to it from colder countries, to pass the winter in the island, and of the frequency of catarrhal affections among the inhabitants; but he strongly insists that genuine consumption is also very common and very fatal. There can however be little doubt, from the concurrent testimony of the majority of observers, that the climate of Madeira is extremely salubrious, and that consumptions, though they may sometimes occur, are comparatively rare.

In the West Indies, it is agreed by all authors, that consumptive affections are almost unknown, and that scrofula in all its forms is uncommon; while the inhabitants of the West Indies, coming into a colder climate, are peculiarly liable to the attacks of these diseases. Dr. Hunter, however, observes, that notwithstanding this exemption in favor of the natives of the West Indies, a residence in this climate appeared to him to be of no manner of advantage to persons who were already affected by incipient consumptions when they arrived there. We cannot doubt the accuracy of this evidence, as far as regards the facts which came immediately under Dr. Hunter's observation; they principally related to the military, who perhaps labored under some peculiar disadvantages: but other practitioners have given much more favorable reports of the events of cases, in which they have made trial of the effect of a residence in this climate; and if we may be allowed to draw any inference from the qualities of a climate, as indicated either by the thermometer, or by its effects on the constitutions of the inhabitants, there can be little doubt that a residence in Bermudas, in a temperate and sheltered part of Jamaica, or in some other of the West-India islands, together with the equable qualities of the sea air, to which the patient must be exposed during the voyage, must present every advantage towards the recovery of a consumptive person, that climate alone can possibly bestow.

In other diseases, the effects of climate are perhaps less exclusively beneficial; although it appears that gouty persons often derive considerable benefit from a residence in the hottest countries, as in the East Indies, or at Ceylon in particular. Dr. Gregory seems to be persuaded that life may be lengthened, and the inconveniences of old age retarded or mitigated, by repeated emigrations into warmer and warmer climates, after the age of 50 or 60, according to circumstances: and he thinks that even posterity may be benefited by an emigration of this kind.

In whatever situation the residence of an invalid may be fixed, it is of no small importance that the aspect and exposure of the house, which he occupies, should be selected with a view to the qualities of climate which he is desirous of obtaining. We have an illustration of the truth of this remark, in an observation recorded by Dr. Carrick, respecting the influenza of 1803. "One of the most open and exposed of the buildings on Clifton-hill is Richmond-terrace, which forms three sides of a parallelogram, fronting respectively the east, south, and west; on the east side, not one family, and scarcely an individual, escaped the complaint; while on the south side, a great majority, both of persons and families, in all other respects similarly circumstanced, escaped it entirely." Such facts as these are among the few which afford solid grounds for medical reasoning; and they deserve the more attention, as they relate to circumstances of continual occurrence, and of perpetual influence on our health and comfort; and in proportion as both the medical and the meteorological sciences become founded on a firmer basis, it cannot be doubted that their beneficial effects will be more and more experienced, as well in the preservation of health, as in the treatment and cure of diseases.

TABLE OF THE ANNUAL MORTALITY

*Of the different Counties of Great Britain, according to the Returns of* 1811.

| | | | |
|---|---|---|---|
| Middlesex | 1 in 36 | Berks | 1 in 53 |
| Kent | 41 | Flint | 53 |
| Warwick | 42 | Glamorgan | 53 |
| Cambridge | 44 | Northumberland | 53 |
| Essex | 44 | Rutland | 53 |
| Surry | 45 | Suffolk | 53 |
| York, E. R. | 47 | Brecon | 54 |
| Huntingdon | 48 | Cumberland | 54 |
| Lancaster | 48 | Westmoreland | 54 |
| Buckingham | 49 | Wilts | 54 |
| Southampton | 49 | Hertford | 55 |
| Mean of England | 49 | Oxford | 55 |
| Chester | 50 | Sussex | 55 |
| Durham | 50 | Bedford | 56 |
| Norfolk | 50 | Derby | 56 |
| Lincoln | 51 | Radnor | 56 |
| York, N. R. | 51 | Dorset | 57 |
| York, W. R. | 51 | Leicester | 57 |
| Denbigh | 52 | Salop | 57 |
| Nottingham | 52 | Devon | 58 |
| Northampton | 52 | Hereford | 58 |
| Somerset | 52 | Mean of Wales | 60 |
| Stafford | 52 | Gloucester | 61 |
| Worcester | 52 | Carmarthen | 62 |

Cornwall

| | | | |
|---|---|---|---|
| Cornwall . . . . | 1 in 62 | Pembroke . . . | 1 in 64 |
| Merioneth . . . . . . | 62 | Carnarvon . . . . | 67 |
| Montgomery . . . . | 63 | Anglesey . . . . . . | 72 |
| Monmouth . . . . | 64 | Cardigan . . . . . . | 73 |

It is obvious, that those counties which contain large manufacturing towns exhibit a mortality wholly independent of their climate, as is exemplified in the case of Warwickshire; while the natural salubrity of others, for instance, Cornwall, is probably rendered more conspicuous by their exemption from sedentary employments.—*Dr. Young's Introduction to Medical Literature.*

